# RNA-seq analysis of shrimp tropomyosin-induced allergic reactions through PI3K/Akt pathway

**DOI:** 10.3389/fnut.2025.1623971

**Published:** 2025-07-09

**Authors:** Yanchu Li, Yuying Yang, Junyao Li, Rui Guo, Zhiqiang Niu, Weicheng Hu, Shucheng Liu, Shuai Wei

**Affiliations:** ^1^College of Food Science and Technology, Guangdong Ocean University, Guangdong Provincial Key Laboratory of Aquatic Product Processing and Safety, Guangdong Province Engineering Laboratory for Marine Biological Products, Guangdong Provincial Engineering Technology Research Center of Seafood, Key Laboratory of Advanced Processing of Aquatic Product of Guangdong Higher Education Institution, Zhanjiang, China; ^2^School of Medicine, Institute of Translational Medicine, Yangzhou University, Yangzhou, China; ^3^Collaborative Innovation Center of Seafood Deep Processing, Dalian Polytechnic University, Dalian, China

**Keywords:** food allergy, tropomyosin, *Litopenaeus vannamei*, mechanism, PI3K/Akt

## Abstract

**Introduction:**

Tropomyosin (TM) is the primary allergen in *Litopenaeus vannamei*, which usually causes allergic reactions that may be health or even life-threatening for consumers. Therefore, exploring the sensitization mechanism is of great significance for the prevention and treatment of tropomyosin allergy.

**Methods and results:**

In this study, TM sensitization models were using Balb/c mice, Caco-2 cells and RBL-2H3 cells to reveal the sensitization effect. The results of ELISA and RT-qPCR showed that TM can exacerbate the allergic reaction by reducing the mRNA expression of tight junction (TJ) proteins (such as ZO-1, claudin-3, Occludin) in the jejunum, destroying the intestinal barrier function, increasing the permeability, and promoting the release of inflammatory factors (such as IL-8, TNF-*α*) and histamine. The pathological results of intestinal tissue sections showed that TM also caused an increase in intestinal inflammatory infiltration in mice. RNA-seq analysis revealed that key genes (CCL2, HSP1A, GM-CSF, etc.) and PI3K/Akt signaling pathway were involved in the sensitization process. *In vitro* experiments were conducted to construct TM sensitized Caco-2 and RBL-2H3 cell models at a dose of 100 mg/mL. The results indicated that TM upregulated the expression of phosphorylated PI3K/ Akt and NFκB pathways in Caco-2 cells, further damaged the TJ structure of intestinal epithelial cells and promoted the release of inflammatory factors. The RBL-2H3 cell degranulation assay indicated that TM could directly stimulate the release of TNF-*α* from mast cells.

**Conclusion:**

The above experimental results indicated that PI3K/Akt signaling pathways play a crucial role in the induction of TM allergic responses, which provides a theoretical basis for the occurrence, development and prevention of TM allergy.

## 1 Introduction

Crustaceans are widely recognized as valuable source of high-quality nutrition (1, 2), particularly due to high content of proteins and fatty acids (3). However, they are also among the nine most common food allergens, with shrimp and crabs being significant contributors (4, 5). The prevalence of crustacean allergy is estimated to affect between 0.5 and 2.5% of the general population. A prior study indicated that the global prevalence of shrimp allergy is 8% in children and 10% in adults (6). Notably, as the most productive species in aquaculture, the increased consumption of *Litopenaeus vannamei* has been associated with an increase in allergy cases [FAO, 2024; (7)]. Tropomyosin (TM) is the primary allergen in *L. vannamei* (8). At present, most of the research on TM sensitization is focused on exploring its allergenic key epitopes (9). Researchers have tried to reduce protein sensitization by modifying known epitopes, but this method is difficult to be widely used in the food processing industry (10). Therefore, researchers have shifted their focus to investigate the sensitization mechanisms of TM, aiming to inhibit the critical signaling pathways using innovative processing techniques to decrease TM sensitization (11).

TM is a long, filamentous protein composed of two identical *α*-helical chains that are intertwined (12, 13). It has an average length of approximately 284 amino acid residues and a molecular mass ranging from 34 to 38 kDa (14, 15). TM allergy is a type of hypersensitivity mediated by Immunoglobulin E (IgE) (16, 17), which possesses B cell epitopes on its surface that could bind to specific antibodies (9). This interaction triggers the immune system to activate B cells, leading to the production of IgE antibodies (18), which then bind to the FcεRI receptor on mast cells or granulocytes (19). This binding induces degranulation and the release of histamine and cytokines (20). Various signaling pathways such as Mitogen-Activated Protein Kinase (MAPK) pathway (21, 22) and the Notch signaling pathway are reported to be involved in the pathophysiology of allergies. The Notch pathway enhanced FcεRI-mediated MAPK phosphorylation, which significantly results in an imbalance of the Th1/Th2 ratio and abnormal immune responses in the body, thereby promoting the process of food allergy (23, 24). It is reported that allergens could activate ERK1/2 initiating Th2 cytokine transcription, ultimately leading to the manifestation of allergic symptoms (25). Xu et al. (26) has found that TM allergy was associated with upregulation of innate (cochlin) and adaptive immunomodulator (IGKV-3). However, at present, no studies have pointed out the key signaling pathways and key genes of TM sensitization, which would be not beneficial for the prevention and treatment of TM allergy. This study combined *in vivo* RNA-seq analysis of signaling pathways with *in vitro* investigations using Caco-2 and RBL-2H3 cell models to elucidate the sensitization mechanism of tropomyosin.

## 2 Materials and methods

### 2.1 Reagents

*L. vannamei* were purchased alive from the Xiashan wholesale Market, Zhanjiang, China. Dithiothreitol (DTT) was obtained from Aladdin (Shanghai, China). 3-(4, 5)-dimethylthiahiazo (−z-y1)-3,5-di-phenytetrazoliumromide (MTT) and 4′,6-diamidino-2-phenylindole (DAPI) were purchased from Sigma (MO, USA). The BCA protein assay kit and Trizol were purchased from Thermo Fisher (MA, USA). ELISA kits for IL-8 and TNF-*α* were acquired from R&D Systems (MN, USA). p-PI3K (Cat:42285), p-Akt (Cat: 4058s), P-NF-kB (Cat: 3033T) and NF-kB (Cat:82425) antibodies were obtained from CST (MA, USA). Polyvinylidene fluoride (PVDF) membranes were purchased from Bio-Rad (CA, USA). Fetal bovine serum and non-essential amino acids were purchased from Gibco (NY, USA). Minimal Essential Medium (MEM) were purchased from HyClone (UT, USA). All other reagents used in this study were of analytical grade.

### 2.2 Animals

Female Balb/c mice (18–22 g) were obtained from SPF Biotechnology Co., Ltd. (Beijing, China) and housed in a specific pathogen-free (SPF) animal facility. All animal experiments were conducted in strict accordance with the guidelines of the Animal Center at Yangzhou University (Approval No. 202408002). The mice were provided with ad libitum access to food and water.

### 2.3 Cell culture

The Caco-2 and RBL-2H3 cell lines were obtained from the China Center for Type Culture Collection (CCTCC). Caco-2 cells were cultured in MEM medium supplemented with 10% (v/v) FBS and 1% antibiotic-antimycotic, in an atmosphere of 5% CO_2_ at 37°C. RBL-2H3 cells were cultured in MEM, supplemented with 10% (v/v) FBS, 1% antibiotic-antimycotic, and 1% NEAA in an atmosphere of 5% CO_2_ at 37°C. After thawing the two cell lines, the third generation can be used for modeling, and they need to be discarded after 30 generations.

### 2.4 Isolation and purification of TM

One gram of shrimp meat was mixed with 1 mL of 0.9% saline solution (w/v). The fat from the shrimp meat was extracted using acetone, at four times the volume of saline, to produce acetone powder. Total protein was subsequently extracted from the shrimp meat using a 0.05 mmol/L Dithiothreitol (DTT) solution. Ten milliliters of DTT solution were added to 1 g of acetone powder, and the mixture was incubated for 4 h before centrifuging to collect the supernatant at 4°C (8,000 × g, 15 min). The pH of the supernatant was adjusted to 4.5 using 1 mol/L HCl and incubated at 20°C for 1 h. The mixture was then centrifuged, and the supernatant was discarded. The resulting precipitate was dissolved in 1 mmol/L NaHCO_3_ and mixed with an equal volume of saturated ammonium sulfate solution. The mixture was allowed to incubate for 4 h at 4°C, and the precipitate was collected by centrifugation. The precipitate was dissolved in 10 mL of 20 mmol/L Tris–HCl. Finally, the crude protein was filtered through a dextran gel G75 column (10 mL column volume) to obtain a high-purity TM.

### 2.5 TM-induced mouse food allergy model

TM-induced mouse food allergy model was established according to previous literature (27). Thirty-two mice were randomly assigned to four groups: phosphate-buffered saline (PBS) group, TM low-dose (6 mg/kg) group, TM medium-dose (12 mg/kg) group, and TM high-dose (24 mg/kg) group. The TM treatment groups received weekly injections for four weeks. From weeks 5 to 8, the protein dose was doubled, and aluminum hydroxide adjuvant was added at a 3:1 ratio of protein to adjuvant. After the final treatment, the mice were euthanized the following day. The thymus and spleen were harvested and weighed to calculate the thymus and spleen indices, and the jejunum was collected for further analysis.

### 2.6 Histological analyses

The intestinal tract was preserved in 4% paraformaldehyde, then dehydrated and embedded in paraffin. It was sectioned into 5 μm slices, placed on slides, and dried in an oven at 75°C for 2 h. The dried sections were dewaxed in xylene twice for 10 min each and then immersed in a series of alcohol solutions (100, 95, 85, 80, 75%) for 5 min each. After staining with hematoxylin–eosin (H&E), periodic acid-Schiff (PAS), and Alcian blue, the sections were rinsed with running water. The sections were then dehydrated using the same gradient of alcohol (75, 80, 85, 95, 100%) for 5 min each and soaked in xylene twice for 10 min each. Micrographs were captured using light microscopy at a wavelength of 555 nm and viewed using CasViewer (Scope AI). Villus length and crypt depth were assessed through H&E staining, while the extent of intestinal inflammatory infiltration was evaluated by analyzing the distribution of goblet cells using PAS and Alcian blue staining.

### 2.7 RNA-seq analysis

The intestines of mice were frozen using liquid nitrogen and then grounded. RNA extraction was performed by adding the tissue to Trizol and incubating on ice for 10 min according to previous literature (28). One hundred microliters of chloroform were added to the Trizol mixture and left on ice for 5 min before centrifugation to remove the supernatant at 4°C (12,000 × g, 15 min). The same volume of isopropyl alcohol was then added, mixed thoroughly, and left at 20°C for 10 min. The supernatant was discarded by centrifugation at 4°C (12,000 × g, 15 min). The RNA was washed with 1 mL of 75% ethanol to remove excess impurities, and the precipitate was collected by centrifugation at 4°C (12,000 × g, 15 min). The centrifuge tubes containing sediment were placed in a clean fume hood to allow the alcohol to evaporate. After drying, the precipitate was dissolved in 30 μL of 0.1% DEPC-treated water. The extracted RNA was examined for purity and quantity using RNA agarose gel, after which the RNA was used for subsequent experiments. Gene expression levels were quantitatively determined using RNA-seq by Expectation–Maximization (RSEM). Differentially expressed genes between the two groups were identified using a rigorous algorithm (DEGseq). Gene Ontology (GO) and pathway annotation, as well as enrichment analysis, were conducted based on the OmicShare.

### 2.8 Cell viability

The cell viability was determined using MTT assay (29). Cells were seeded into 96-well plates at a density of 3 × 10^4^ cells per well and cultured overnight (37°C, 5% CO_2_). The cells were treated with various concentrations (25, 50, 100 μg/mL) of TM for 24 h. Following treatment, 100 μL of MTT solution (0.5 mg/mL) was added, and the cells were incubated at 37°C for 4 h. Subsequently, MTT stop solution was added, and the cells were incubated overnight. The absorbance was measured at 550 nm, and the cell viability was calculated.

### 2.9 IL-8, TNF-*α* and histamine release

The eyeball blood of the mouse was centrifuged at 4°C (2,500 rpm, 5 min), and the supernatant was stored at −80°C for future use. IL-8, histamine in mouse serum, and TNF-α in the supernatant of RBL-2H3 cells were detected using commercial ELISA kits (D8000C; E-EL-0032; MTA00B-1; R&D Systems), following the manufacturer’s instructions.

### 2.10 Real-time qRT-PCR

RNA was extracted using the Trizol method, and cDNA was synthesized using a reverse transcription kit (Takara, Japan). The samples were stored at −20°C for future use. Specific primers listed in Table 1 were designed with SnapGene 4.2.4 and their specificities were confirmed by blasting against the genome using http://www.ncbi.nlm.nih.gov/tools/primer-blast. All primers for qRT-PCR were synthesized by Shanghai Bioengineering Co., Ltd. (Shanghai, China). The relative expression levels were evaluated using the ^2 − ΔΔ^Ct method.

**Table 1:** Primer sets for RT-qPCR

Primers	Sequences (5’-3’)	
Caludin-3*	Forward	TCATCGTGGTGTCCATCCTGCT
	Reverse	AGAGCCGCCAACAGGAAAAGCA
Caludin-1	Forward	AGCACCGGGCAGATACAGT
	Reverse	GCCAATTACCATCAAGGCTCG
ZO-1*	Forward	TGGTGTCCTACCTAATTCAACTC
	Reverse	CGCCAGCTACAAATATTCCAACA
ZO-1	Forward	ACCCGAAACTGATGCTGTGGATAG
	Reverse	AAATGGCCGGGCAGAACTTGTGTA
Occludin*	Forward	ACAGAGCAAGATCACTATGAGACA
	Reverse	TGTTGATCTGAAGTGATAGGTGGA
Occludin	Forward	ATGTCCGGCCGATGCTCTC
	Reverse	TTTGGCTGCTCTTGGGTCTGTAT
OCLN*	Forward	TTGGATAAAGAATTGGATGAC
	Reverse	ACTGCTTGCAATGATTCTTCT
JAM-1*	Forward	AACACACTGGGACATACACTT
	Reverse	CGATGAGCTTGACCTTGACCT
IGHV3-23	Forward	GAGGTGCAGCTGTTGGAGTC
	Reverse	TGAGGAGACAGTGACCAGGG
HSP1A	Forward	GGTGGTGCAGTCCGACATG
	Reverse	TTGGGCTTGTCGCCGT
CD36	Forward	TGGTCAAGCCAGCTAGAAA
	Reverse	TCCCAAGTAAGGCCATCTC
GM-CSF	Forward	GGCTAAGGTCCTGAGGAGGAT
	Reverse	ACCTCTTCATTCAACGTGACA
CCL2	Forward	TCCCAATGAGTAGGCTGGAG
	Reverse	TCTGGACCCATTCCTTCTTG
GAPDH*	Forward	TCCACCACCCTGTTGCTGTA
	Reverse	ACCACAGTCCATGCCATCAC
GAPDH	Forward	CCATCTTCCAGGAGCGAGAC
	Reverse	GGTCATGAGCCCTTCCACAA

* This symbol indicates the primers are derived from the human homologous, while the others are from the murine sequence.

### 2.11 Immunofluorescence staining

Caco-2 cells were seeded into a 12-well plate at a density of 5 × 10^5^ cells per well. After 24 h, the cells were treated with 100 μg/mL TM for 12 h. Subsequently, the cells were incubated overnight with 300 μL of primary antibodies: occludin (1:2500) and claudin-1 (1:250). Following this, 300 μL of goat anti-rabbit Alexa 488 and goat anti-mouse Alexa 555 (1:1200) secondary antibodies were incubated for 1 h. Ten-microliters of 1 mg/mL 4′,6-Diamidino-2′-phenylindole (DAPI) solution were added before sealing the wells. Images were captured using fluorescence microscopy Nikon Ts2.

### 2.12 Western blotting

The tissues were ground with liquid nitrogen and then lysed in lysis buffer containing protease inhibitors and the content was quantified using a BCA kit. The proteins were separated by 10% w/v polyacrylamide gel electrophoresis and transferred onto PVDF membranes. The membranes were then blocked and incubated overnight at 4°C with 1 mL of primary antibodies against p-PI3K, p-Akt, PI3K, and Akt (diluted 1:1000). Samples were rinsed three times with 1 × Tris Buffered Saline with Tween 20 (TTBS) for 10 min. After washing, HRP-conjugated secondary antibodies were applied at a dilution of 1:2500. Protein bands were visualized using an ECL detection kit and captured with a Tanon 5,200 multi gel imaging system.

### 2.13 Statistical analysis

All analyses were carried out in triplicate, and the results were expressed as means ± standard deviation (SD). The graphpad prism software 10.1.2 was utilized for plotting and conducting one-way analysis of variance. *p* < 0.05 was considered statistically significant.

## 3 Results and discussion

### 3.1 Phenotypic characteristics of TM-sensitized mice

**Figure 1:**
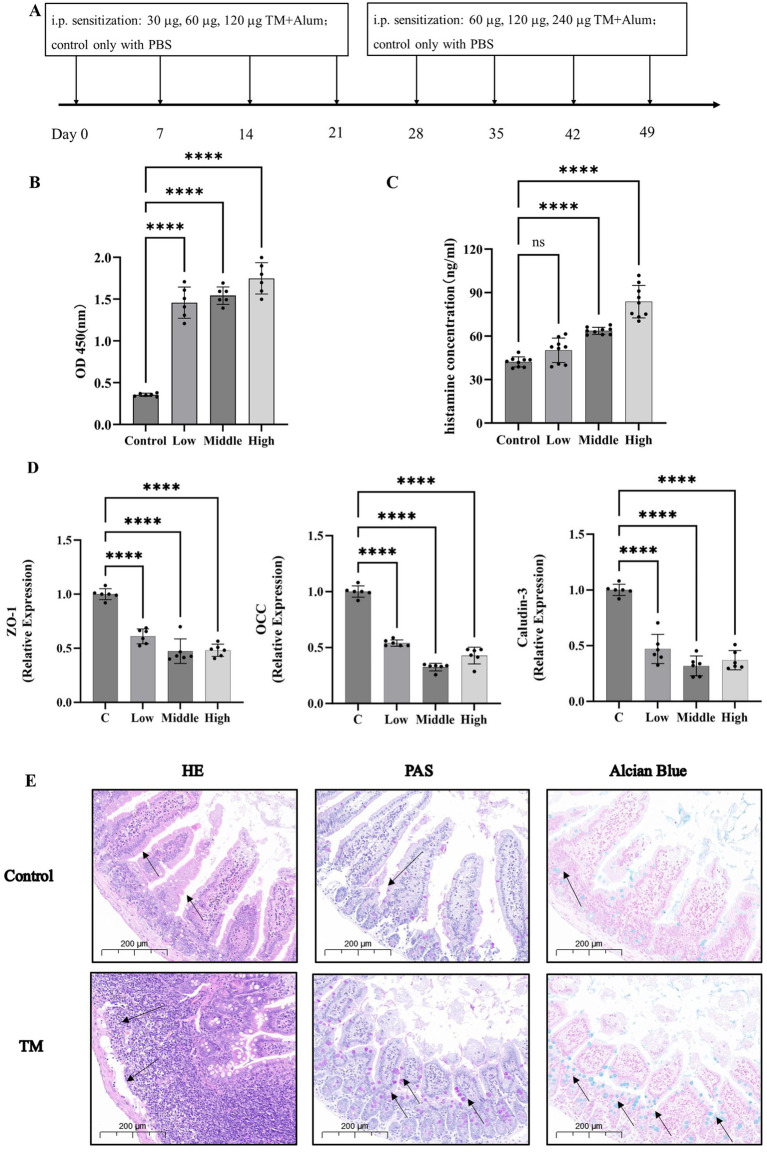
TM leads to the increase of serum inflammatory cytokines and the destruction of the intestinal barrier. **(A)** Mice allergy test protocol. **(B)** Serum levels of IgE in mice. **(C)** Histamine levels in serum of mice. **(D)** The relative expression of TJ proteins in mouse jejunum. **(E)** H&E, PAS, Alcian Blue of the intestinal of mice. *p < 0.05; **p < 0.01; ***p < 0.001; ****p < 0.0001.

Strategies for experimental design in mice were showed in Figure 1A. Firstly, the construction of the sensitization model is generally divided into two stages, namely the sensitization stage and the excitation stage. The sensitization stage is when the allergen acts on the body for the first time through certain pathways. The body develops a specific immunity to this substance and reaches a certain level, thus being in a hypersensitive state. The excitation stage refers to the specific pathological immune response that occurs when the body is exposed to the substance again. After the body first comes into contact with an allergen, there is an incubation period. Generally speaking, the incubation period is relatively short, sometimes taking 3 to 4 weeks or even longer. Referring to the mouse sensitization model constructed by Zhang et al. (30) and Luo et al. (31), the first four weeks were selected as the sensitization stage in this study. During the excitation stage, a doubled dose was injected into the mice. The mice would show changes such as scratching, rough fur, and weight loss. The symptoms of the mice were observed after each injection, and the weight of the mice was measured the day after the injection. At the eighth week, it was found that most of the mice had lost weight and were restless. According to the symptoms of the mice, they were sacrificed after injection at the eighth week.

After IgE binding of antibodies in serum, the FCεR receptor stimulates mast cells and basophils to degranulation, releasing histamine and other substances to promote allergy (32). In this experiment, indirect ELISA was used to detect the IgE binding ability of TM. As shown in Figure 1B, the IgE binding ability of TM protein in the serum of TM group was 1.3–1.8, which was significantly higher than that of the control group (0.35) (*p* < 0.0001). This indicates that IgE binding is active in allergic mice, thus promoting the occurrence of allergic reactions. Histamine levels mediate allergic reactions, increase capillary permeability, and act as a neurotransmitter to regulate pain and itchy nerves, which is an important indicator to evaluate the severity of allergies (33). Ando et al. (34) study found that histamine can interact with some IgE molecules to amplify intestinal inflammation, this experiment, the TM mice after high dose sensitization of histamine release quantity increased from 42 ng/mL to 85 ng/mL (Figure 1C), which is similar to his research.

Previous studies have proposed the “epithelial barrier hypothesis” of allergy, which suggests that the intestinal barrier is closely related to food allergy (35–37). Mice with a protected intestinal barrier had less infiltration of neutrophils in the gut and fewer food allergies (38). In contrast, downregulation of TJ proteins expression and increased epithelial permeability led to a significant increase in allergy (39). In this study, RT-qPCR and intestinal histopathological sections were used to further elaborate the effects of TM on the intestinal tract of mice. The results of RT-qPCR were shown in Figure 1D. After TM treatment, mRNA levels of TJ proteins ZO-1, OCC, and Claudin-3 in mouse jejunum were significantly decreased (*p* < 0.0001). In general, mRNA levels are positively correlated with protein levels. Therefore, the results indicated that TJ in the gut of mice is reduced. These findings align with Jia et al. (35). However, different from his focus only on occludin, this study focused on occludin, Claudin and the perimembrane protein family (ZO proteins), demonstrating that the reduction of TJ is related to a variety of proteins from a more comprehensive perspective, which provides a new perspective for the damage of intestinal barrier in allergic mice.

Intestinal histopathological sections are the most intuitive way to diagnose intestinal diseases (40). Different from previous pathological sections, this study not only used H&E to pay attention to the changes in villus length and inflammatory infiltration of mouse jejunum tissue, but also used PAS and Alcian blue staining to observe the changes in the number of goblet cells. The staining results were shown in Figure 1E. Compared with the control group, H&E results showed that the length of jejunal villi was significantly shortened, and inflammatory infiltration was increased in the TM group. PAS and Alcian blue results showed that the number of goblet cells in the jejunum of the TM group was significantly increased. Intestinal villi are related to normal physiological functions such as nutrient absorption (41). The shortened villi showed that TM treatment disrupted the normal absorption and metabolism of intestinal substances, promoted the occurrence of intestinal inflammation and accelerated the occurrence of allergic reactions. Goblet cells respond to cytokines during intestinal inflammation and proliferate to secrete more mucus (42). Therefore, the increase in the number of goblet cells in the TM group indicates that TM can promote the release of intestinal inflammatory factors in mice, thus promoting intestinal inflammation, which is also consistent with the results in Figure 1C.

These showed that TM treatment could increase the inflammatory factors in the serum of mice, leading to pruritus and other allergic phenomena in mice. In addition, TM treatment also resulted in decreased expression of tight binding proteins in the digestive tract of mice, so that more inflammatory factors could pass through the intestinal wall into the body, and intestinal inflammatory infiltration increased, which also aggravated allergic reactions.

### 3.2 Analysis of the jejunum transcriptome following sensitization and challenge to TM in mice

**Figure 2:**
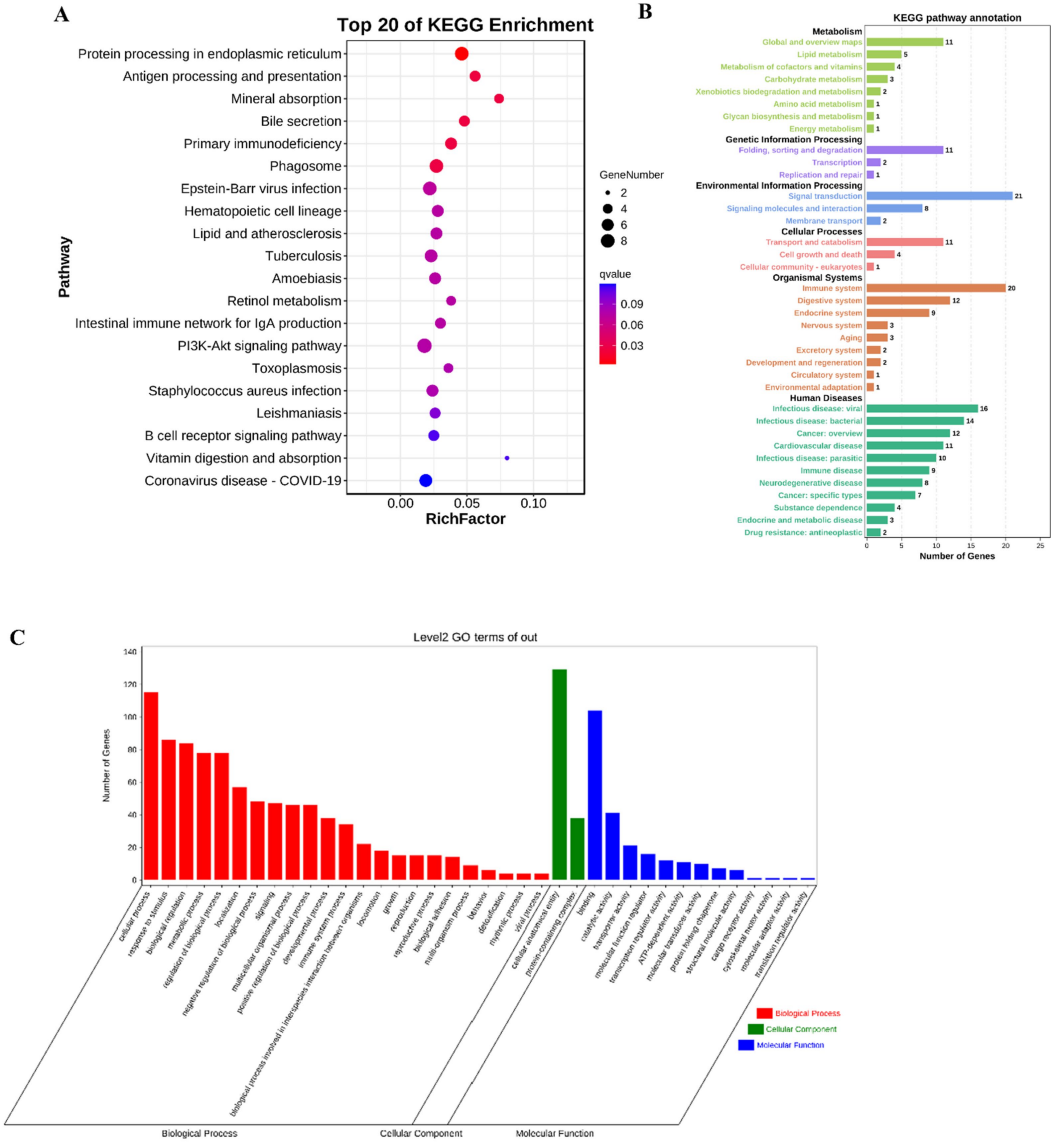
DEG identification and KEGG enrichment information analyses between the C and TM groups. **(A)** The KEGG bubble maps of 20 pathways with the highest differential gene enrichment in mouse jejunum were mapped by RNA-seq technique. **(B)** KEGG pathway annotation diagram. **(C)** GO hierarchical diagram.

One of the most common applications of RNA-seq data is Differentially expressed genes (DEGs) analysis (43). In this study, the transcriptome changes of mouse jejunum sensitized by tropomyosin were detected, and more than 200 DEGs were selected. The pathway data of DEGs were sorted out, and 20 signaling pathways with the highest degree of enrichment were finally obtained (Figure 2A). All enrichment pathways are summarized and classified according to secondary structure, they are more enriched in the immune system and signal transduction (Figure 2B). Fisher algorithm was used to analyze the enrichment of biological process, cellular component and molecular function of differential genes, which showed the GO nodes of gene enrichment more intuitively. The results show that cellular process enriched the most significant differential genes (Figure 2C).

### 3.3 Exploration of key sensitizing genes and pathways in the jejunum of mice after TM sensitization and challenge

**Figure 3:**
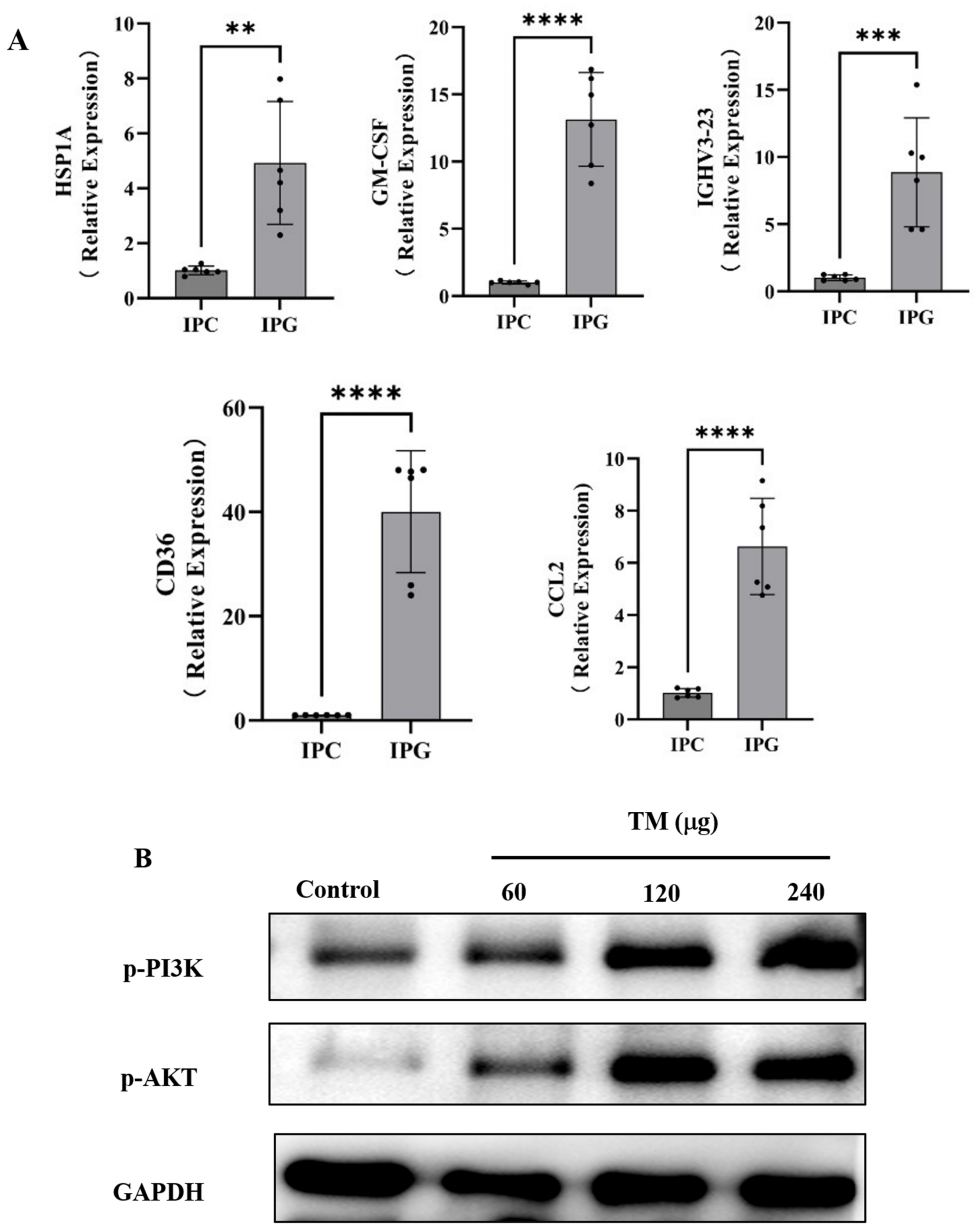
TM causes hypersensitivity in mice by regulating the PI3K/Akt signaling pathway. **(A)** Relative expression levels of HSP1A, GM-CSF, IGHV3-23, CD36 and CCL2 in mouse jejunum. **(B)** Western blot analysis of p-PI3K, p-Akt expression in mice intestinal after different treatments. *p < 0.05; **p < 0.01; ***p < 0.001; ****p < 0.0001.

According to the results of DEGs, Gene Ontology (GO) enrichment results and Kyoto Encyclopedia of genes and Genomes (KEGG) enrichment results, combined with literature review, five DEGs were finally obtained, including Heat Shock Protein Family A (HSP1A), Granulocyte-Macrophage Colony-Stimulating Factor (GM-CSF), Immunoglobulin Heavy Variable 3–23 (IGHV3-23) (44), Platelet glycoprotein 4 (CD36), and C-C motif chemokine ligand 2 (CCL2). When CCL2 binds to the receptor Chemokine receptor 2 (CCR2), it will activate PI3K/Akt and MAPK/p38. CCL2 participates in calcium ion influx by activating these signaling pathways and plays a crucial role in immune defense (45). CD36 has the function of signal transduction. In response to extracellular signals, CD36 assembles and interacts with other membrane receptors to form different signal complexes. The signal complex then transmits the signal to various downstream effect molecules, including MAPK and AMPK. The activation of these effector factors leads to the generation of ROS and the activation of NF-κB, resulting in different cellular responses, such as inflammation, immune activation, and cell death (46). HSP1A stimulates neutrophils to release chemokines CD14 and TLR2. The activation of TLR2 upregulates the level of IFN-*γ* in the body, thereby promoting allergic reactions (47). GM-CSF particularly promotes the proliferation and maturation of neutrophils, and these cells can produce pro-inflammatory cytokines such as TNF-*α*, IL-6 and IL-12 (48). To detect the relationship between the changes of these five key genes and TM sensitization, RT-qPCR was used to reveal the changes of mRNA expression levels of key genes in the jejunum of mice after TM treatment. The results showed that the expressions of CCL2, HSP1A, GM-CSF, IGHV3-23 and CD36 in the TM group were significantly up-regulated (Figure 3A), which was consistent with the results of RNA-seq, indicating that CCL2, HSP1A, GM-CSF, IGHV3-23 and CD36 were the key genes of TM sensitization. This provided a new target for the prevention and treatment of TM allergy.

As shown in Figure 2A, PI3K is one of the important enriched pathways during sensitization. It is reported that the PI3K signaling pathway is involved in OVA-induced food allergy models (49). However, there is no clear research showing that TM sensitization is related to PI3K. Western blot, which can use specific antibodies to detect the expression level and post-translational modification status of target proteins, was used in this paper to reveal changes in the expression of proteins related to the PI3K/Akt signaling pathway. As shown in Figure 3B, compared with the control group, the expression of p-PI3K, p-Akt, Akt protein in the TM group showed a dose-dependent increase, reaching the maximum at 240 μg/mL, indicating that TM can up-regulate the expression of PI3K/Akt signaling pathway protein, thus accelerate the occurrence of inflammation.

### 3.4 TM induces inflammation RBL-2H3 cells

**Figure 4:**
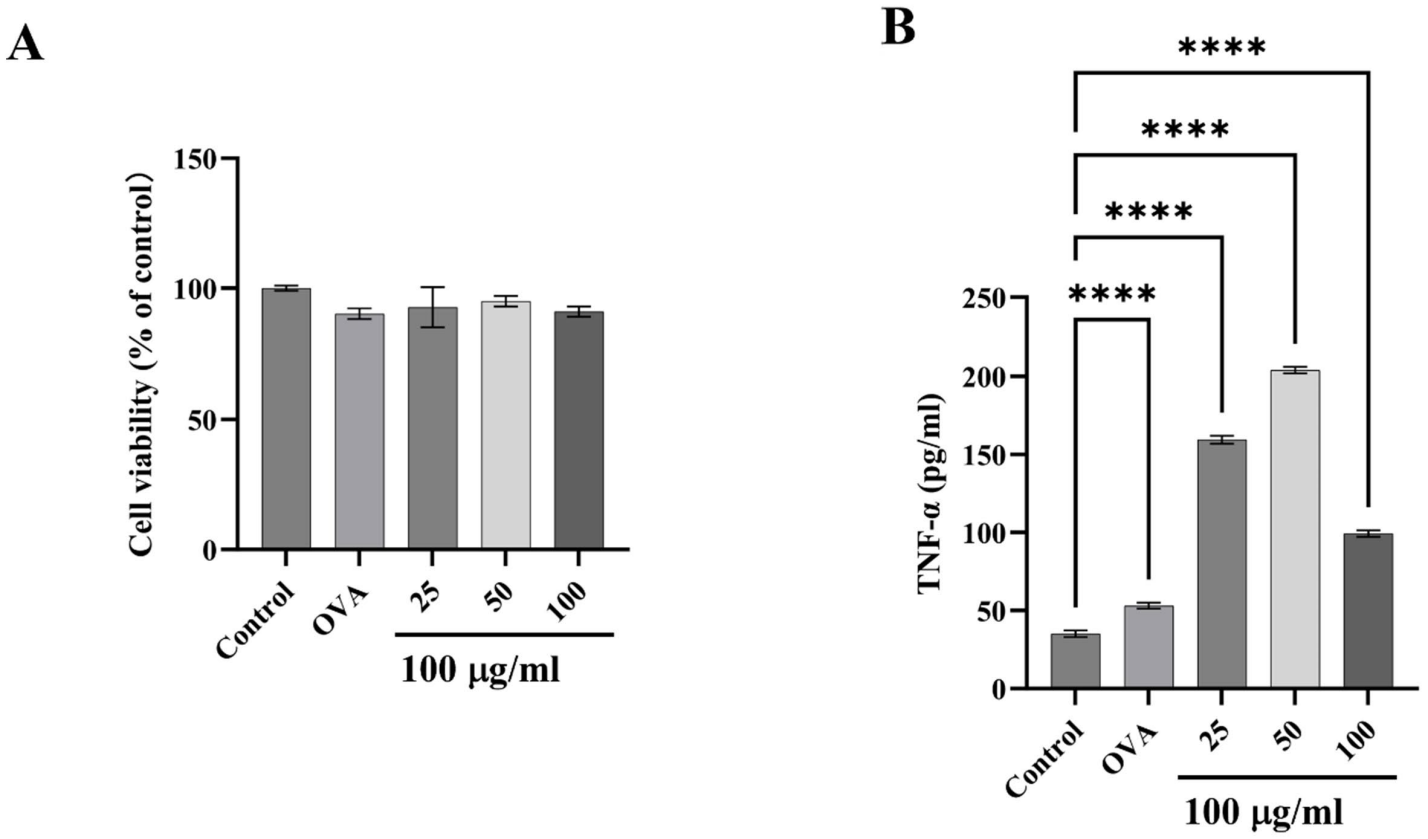
Effect of TM on inflammatory release of RBL-2H3. **(A)** Cell viability. **(B)** TNF-α level in the supernatant of RBL-2H3 cell culture. *p < 0.05; **p < 0.01; ***p < 0.001; ****p < 0.0001.

RBL-2H3 cells are extensively utilized in allergy and immunology research (50), which exhibit characteristics of both mucosal mast cells and basophils, playing a crucial role in simulating cell degranulation during allergic responses (51). Cell viability test data showed that the survival rate of RBL-2H3 remained higher than 90% under high dose of TM treatment, indicating that TM treatment had little effect on cell viability (Figure 4A). RBL-2H3 can also be activated by IgE, leading to degranulation and release of cytokines such as TNF-*α* (52). In this study, the amount of TNF-α released by RBL-2H3 after different doses of TM was measured by indirect ELISA. As shown in Figure 4B, compared with group C, the release of TNF-α increased from 40 pg./mL to 200 pg./mL after medium dose TM treatment, indicating that TM treatment could promote cell degranulation and produce inflammatory factors, thereby accelerating allergy.

### 3.5 TM induces impaired tight junctions in Caco-2 cells

**Figure 5:**
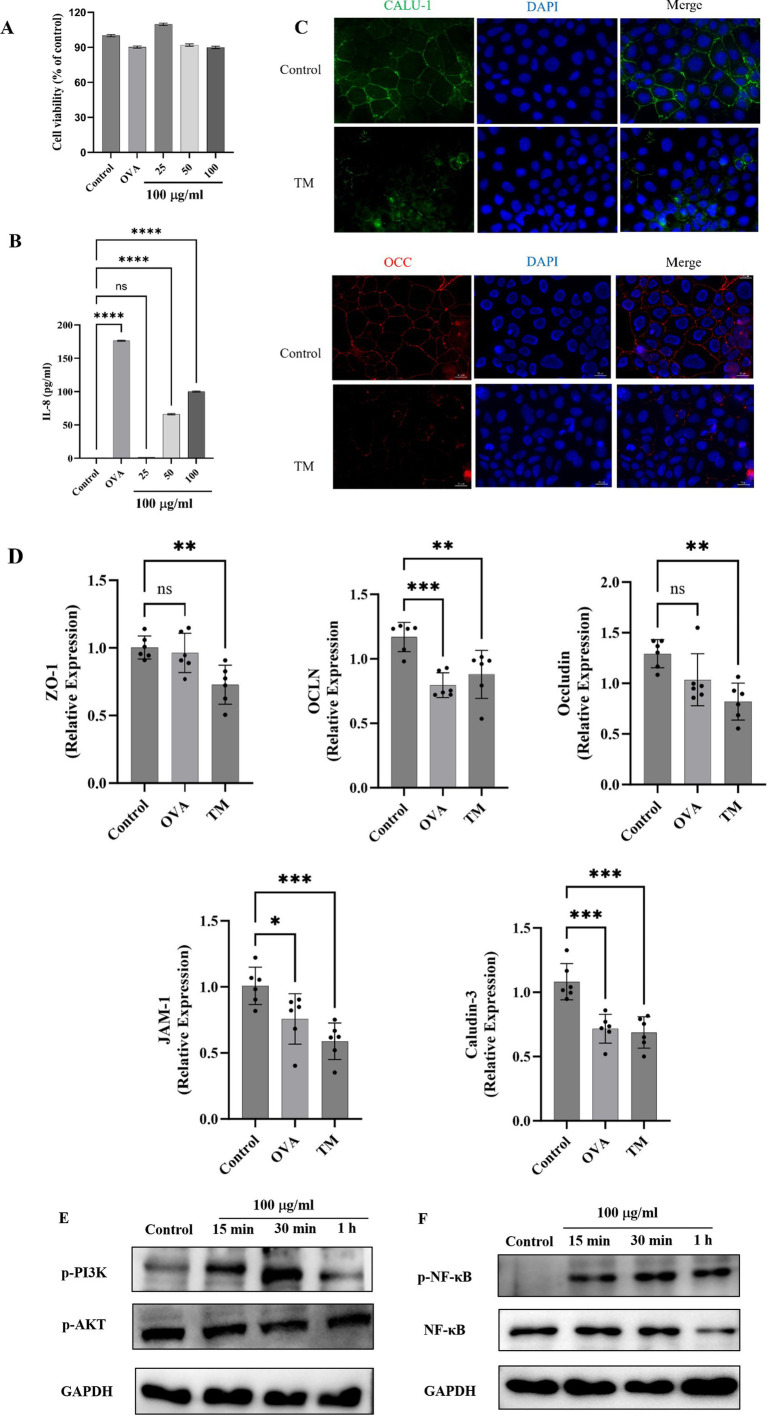
TM activates PI3K/Akt/NF-κB pathway of Caco-2 and decreases TJ proteins expression. **(A)** Cell viability. **(B)** IL-8 level in Caco-2 cell culture supernatant. **(C)** Effect of TM treatment on TJ proteins Occludin and Claudin in Caco-2 cells. **(D)** mRNA relative expression of ZO-1, Occludin, OCLN, JAM-1 and Claudin-3 in Caco-2 cells. **(E)** Western blot analysis of p-PI3K and p-Akt expression in Caco-2 after different treatments. **(F)** Western blot analysis of P-NF-κB and NFκB expression in Caco-2 after different treatments. * *p* < 0.05; ** *p* < 0.01;*** *p* < 0.001;**** *p* < 0.0001; ns *p* > 0.05.

Cell viability is an important index to determine the overall health of cells and optimize experimental conditions (53). As shown in Figure 5A, the cell viability of Caco-2 cells after treatment with different concentrations of TM was all higher than 90%, indicating that TM was safe for Caco-2 cells within the concentration of 100 μg/mL. Therefore, 100 μg/mL was used to treat cells in the subsequent experiment. IL-8 is a typical pro-inflammatory factor, which can target neutrophils in intestinal inflammation, promote degranulation, and aggravate pro-inflammatory response (54). In this experiment, IL-8 released from Caco-2 significantly increased after treatment with medium and high doses of TM (*p* < 0.0001) (Figure 5B), indicating that TM could enhance the release of pro-inflammatory factor IL-8, thus promoting intestinal inflammation and exacerbating allergic reactions. This indicates that TM allergy might be alleviated by inhibiting IL-8.

TJ is an important component to maintain the normal physiological function of the intestinal barrier and an important index to evaluate allergenic sensitization (55). In this section, immunofluorescence and RT-qPCR were used to further reveal the relationship between TM treatment and reduced TJ proteins expression. Immunofluorescence results are shown in Figure 5C. Compared with the control group, TM treatment reduced the fluorescence intensity of Caludin-3 and Occludin, decreased the continuity of TJ proteins, increased the permeability of the intestinal barrier, and caused more inflammatory factors to pass through the barrier into the blood, promoting the occurrence of systemic inflammatory response. TJ proteins consists of occludin and members of the claudin family and junctional adhesion protein (JAM). To explore whether other components are affected (56), qRT-PCR was adopted and the results showed that TM treatment significantly reduced mRNA expression of ZO-1, JAM-1, claudin-3, occludin, and OCLN related to cell TJ proteins (*p* < 0.01) (Figure 5D).

Ma et al. (39) first found that the increase of Caco-2 permeability was related to the change of ZO-1 protein binding location and down expression, and the change of ZO-1 was mediated by the activation of NFκB. Later, Fuentes et al. (57); Roselli et al. (58) and Song et al. (59) found that Caco-2 would release NFκB when stimulated by specific signals. NFκB subsequently translocated to the nucleus, ultimately enhanced the inflammatory response of Caco-2 cells. Compared with the control group, TM treatment for 15 min can significantly increase the expression of P-NFκB, and the expression level of P-NFκB tends to be consistent after 15 min, while TM treatment has little effect on NFκB (Figure 5E), which indicated that TM treatment could lead to NFκB activation and rapid nuclear entry, aggravating the allergic phenomenon (Figure 5).

PI3K/ Akt is the upstream pathway of NFκB. Akt activates the Inhibitor of Kappa B kinase (IKK) directly or indirectly through other molecules, promoting the phosphorylation and degradation of Inhibitor of NF-κB (IΚB), which releases NF-κb into the nucleus and activates transcription of target genes. Zhao et al. (60) determined that PI3K/Akt could promote allergy in combination with NFκB through network pharmacology and transcriptomic analysis. Wu et al. (49) found that PI3K signaling pathway was involved in sensitizing response by establishing an OVA-induced food allergy model. In this study, in order to explore whether TM can cause changes in PI3K/Akt signaling pathway, western blot was used to detect proteins related to PI3K/Akt signaling pathway. As shown in Figure 5F, p-PI3K increased significantly at 30 min, while Akt significantly decreased at 15 min, indicating that both PI3K signaling pathway and Akt signaling pathway were involved in the TM sensitization process and promoted the generation of allergies.

## 4 Conclusion

This study examined the role of PI3K/Akt in the context of TM allergy. The findings suggest that TM modulates the expression of intestinal TJ proteins via the PI3K signaling pathway in murine models, up-regulating key genes CCL2, HSP1A, etc., leading to increased intestinal permeability and a subsequent heightened risk of allergic responses. In cell models, TM can reduce the TJ between Caco-2 cells, increase the degranulation of RBL-2H3 cells, release more inflammatory factors, and promote allergy. This discovery provides a new idea and a new target for the prevention and treatment of TM allergy. In the future, the key structure of TM sensitization can be further explored, the processing method of TM desensitization can be innovated, and effective drugs can be developed to treat TM sensitization, to reduce the incidence of TM allergy.

## Data Availability

The original contributions presented in the study are included in the article materials, further inquiries can be directed to the corresponding author.

## References

[1] Li X Han T Zheng S Wu G. Nutrition and functions of amino acids in aquatic crustaceans. Adv Exp Med Biol. (2021) 1285:169–98. doi: 10.1007/978-3-030-54462-1_9, PMID: 33770407

[2] Nanda PK Das AK Dandapat P Dhar P Bandyopadhyay S Dib AL. Nutritional aspects, flavour profile and health benefits of crab meat based novel food products and valorisation of processing waste to wealth: a review. Trends Food Sci Tech. (2021) 112:252–67. doi: 10.1016/j.tifs.2021.03.059

[3] Dong FJ Bi YZ Hao J Liu SJ Yi WG Yu WJ. A new comprehensive quantitative index for the assessment of essential amino acid quality in beef using Vis-NIR hyperspectral imaging combined with LSTM. Food Chem. (2024) 440:138040. doi: 10.1016/j.foodchem.2023.138040, PMID: 38103505

[4] Khora SS. Seafood-associated shellfish allergy: a comprehensive review. Immunol Investig. (2016) 45:504–30. doi: 10.1080/08820139.2016.1180301, PMID: 27404324

[5] Wai CY Leung NY Leung AS Ngai SM Pacharn P Yau YS. Comprehending the allergen repertoire of shrimp for precision molecular diagnosis of shrimp allergy. Allergy. (2022) 77:3041–51. doi: 10.1111/all.15370, PMID: 35567339 PMC9795902

[6] Chudoba A Żebrowska A Sybilski AJ. Tree nut allergy in children—what do we know? —a review. Nutrients. (2024) 16:3978. doi: 10.3390/nu16233978, PMID: 39683372 PMC11643764

[7] Naidu BC Xavier KA Sahana MD Landge AT Jaiswar AK Shukla SP. Temporal variability of microplastics in shrimp (*Litopenaeus vannamei*), feed, water and sediments of coastal and inland culture ponds. Sci Total Environ. (2025) 959:178173. doi: 10.1016/j.scitotenv.2024.178173, PMID: 39709842

[8] Cheng J-H Wang H Sun D-W. An overview of tropomyosin as an important seafood allergen: structure, cross-reactivity, epitopes, allergenicity, and processing modifications. Compr Rev Food Sci Food Saf. (2022) 21:127–47. doi: 10.1111/1541-4337.12889, PMID: 34954871

[9] Xu LL Chen J Sun LR Gao X Lin H Ahmed I. Analysis of the allergenicity and B cell epitopes in tropomyosin of shrimp (*Litopenaeus vannamei*) and correlation to cross-reactivity based on epitopes with fish (*Larimichthys crocea*) and clam (*Ruditapes philippinarum*). Food Chem. (2020) 323:126763. doi: 10.1016/j.foodchem.2020.126763 32334299

[10] Sun-Waterhouse D Zhao M Waterhouse GI. Protein modification during ingredient preparation and food processing: approaches to improve food processability and nutrition. Food Bioprocess Technol. (2014) 7:1853–93. doi: 10.1007/s11947-014-1326-6

[11] Veerasamy T Eugin Simon S Tan KO. Emerging strategies for sensitization of therapy resistant tumors toward cancer therapeutics by targeting the Bcl-2 family, TGF-β, Wnt/β-catenin, RASSF and miRNA regulated signaling pathways. Int J Biochem Cell Biol. (2021) 137:106016. doi: 10.1016/j.biocel.2021.106016 34082133

[12] Astbury WT Reed R Spark LC. An X-ray and electron microscope study of tropomyosin. Biochem J. (1948) 43:282–7. doi: 10.1042/bj0430282, PMID: 16748402 PMC1274681

[13] Whitby FG Phillips GN. Crystal structure of tropomyosin at 7 angstroms resolution. Proteins. (2000) 38:49–59. 10651038

[14] Costa J Villa C Verhoeckx K Cirkovic-Velickovic T Schrama D Roncada P. Are physicochemical properties shaping the allergenic potency of animal allergens? Clinic Rev Allerg Immunol. (2022) 62:1–36. doi: 10.1007/s12016-020-08826-1, PMID: 33411319

[15] Loo JA Edmonds CG Smith RD. Tandem mass spectrometry of very large molecules: serum albumin sequence information from multiply charged ions formed by electrospray ionization. Anal Chem. (1991) 63:2488–99. doi: 10.1021/ac00021a018, PMID: 1763807

[16] Poulsen LK Hummelshoj L. Triggers of IgE class switching and allergy development. Ann Med. (2007) 39:440–56. doi: 10.1080/07853890701449354, PMID: 17852040

[17] Sicherer SH Sampson HA. Food allergy: epidemiology, pathogenesis, diagnosis, and treatment. J Allergy Clin Immunol. (2014) 133:291–308. doi: 10.1016/j.jaci.2013.11.020 24388012

[18] Kanagaratham C El Ansari YS Lewis OL Oettgen HC. IgE and IgG antibodies as regulators of mast cell and basophil functions in food allergy. Front Immunol. (2020) 11:603050. doi: 10.3389/fimmu.2020.603050, PMID: 33362785 PMC7759531

[19] Nagata Y Suzuki R. FcεRI: a master regulator of mast cell functions. Cells. (2022) 11:622. doi: 10.3390/cells11040622, PMID: 35203273 PMC8870323

[20] Befus AD Mowat C Gilchrist M Hu J Solomon S Bateman A. Neutrophil Defensins induce histamine secretion from mast cells: mechanisms of action. J Immunol. (1999) 163:947–53. doi: 10.4049/jimmunol.163.2.947, PMID: 10395691

[21] Kim EG Kim MN Hong JY Lee JW Kim SY Kim KW. Chitinase 3-like 1 contributes to food allergy via M2 macrophage polarization. Allergy Asthma Immunol Res. (2020) 12:1012–28. doi: 10.4168/aair.2020.12.6.1012, PMID: 32935492 PMC7492506

[22] Wang JJ Song RL Lan RX Hao MZ Liu GR Liu MM. Peanut allergen induces more serious allergic reactions than other allergens involving MAPK signaling pathways. Food Funct. (2022) 13:8818–28. doi: 10.1039/d2fo00777k, PMID: 35920097

[23] Jiang SS Han SW Chen JY Li XJ Che HL. Inhibition effect of blunting notch signaling on food allergy through improving TH1/TH2 balance in mice. Ann Allergy Asthma Immunol. (2017) 118:94–102. doi: 10.1016/j.anai.2016.10.024, PMID: 28007091

[24] Tang XL Meng XR Wang HP Wang T Li Q Jiang SS. Egg allergy was alleviated after baking and frying cooking by weakening Jagged2-notch induced Th2 immunity in a mice model. Eur Food Res Technol. (2022) 248:917–27. doi: 10.1007/s00217-021-03938-0

[25] Li Y Kulis M Pons L Zhong X-P Burks AW. Peanut allergen Ara h 2-specific T cells are activated via Ras-Erk MAP kinase pathway signalling and identified by CD154 expression. Food Agric Immunol. (2011) 22:335–44. doi: 10.1080/09540105.2011.579591

[26] Xu LL Zhang HW Lin H Mei ZX Wen YQ Zhao JL. SWATH-MS-based proteomics reveals functional biomarkers of Th1/Th2 responses of tropomyosin allergy in mouse models. Food Chem. (2022) 383:132474. doi: 10.1016/j.foodchem.2022.132474 35189446

[27] Nakamura A Watanabe K Ojima T Ahn D-H Saeki H. Effect of Maillard reaction on Allergenicity of scallop tropomyosin. J Agric Food Chem. (2005) 53:7559–64. doi: 10.1021/jf0502045, PMID: 16159186

[28] Toni LS Garcia AM Jeffrey DA Jiang X Stauffer BL Miyamoto SD. Optimization of phenol-chloroform RNA extraction. MethodsX. (2018) 5:599–608. doi: 10.1016/j.mex.2018.05.011, PMID: 29984193 PMC6031757

[29] Zhou X Niu ZQ Wu HF Chen B Li JY Qi LY. Novel β-γ′ type lignans from cultivated *Notopterygium incisum* and their potential anti-inflammatory activity. Ind Crop Prod. (2025) 229:120944. doi: 10.1016/j.indcrop.2025.120944

[30] Zhang Z Li X-M Xiao H Nowak-Wegrzyn A Zhou P. Insight into the allergenicity of shrimp tropomyosin glycated by functional oligosaccharides containing advanced glycation end products. Food Chem. (2019) 302:125348. doi: 10.1016/j.foodchem.2019.125348 31442704

[31] Luo C Chen G Ahmed I Sun L Li W Pavase TR. Immunostimulatory and allergenic properties of emulsified and non-emulsified digestion products of parvalbumin (*Scophthalmus maximus*) in RBL-2H3 cells and BALB/c mouse models. Food Funct. (2021) 12:5351–60. doi: 10.1039/d1fo00575h, PMID: 33982680

[32] Chang Y-Y Hsu W-H Pan T-M. Monascus secondary metabolites monascin and ankaflavin inhibit activation of RBL-2H3 cells. J Agric Food Chem. (2015) 63:192–9. doi: 10.1021/jf504013n, PMID: 25525886

[33] Mensching D. Volmer P.A. (2007). Chapter 9 - neurotoxicity. Academic Press, Oxford, pp. 129–144.

[34] Ando T Kashiwakura J Itoh-Nagato N Yamashita H Baba M Kawakami Y. Histamine-releasing factor enhances food allergy. J Clin Invest. (2017) 127:4541–53. doi: 10.1172/JCI96525, PMID: 29130935 PMC5707161

[35] Jia SQ Lin SY Liu QZ Wang SY Chen XH Sun N. Unveiling the impact of dietary components on tropomyosin-induced anaphylaxis: analysis from the perspective of intestinal barrier. Food Biosci. (2024) 62:105254. doi: 10.1016/j.fbio.2024.105254

[36] Samadi N Klems M Untersmayr E. The role of gastrointestinal permeability in food allergy. Ann Allergy Asthma Immunol. (2018) 121:168–73. doi: 10.1016/j.anai.2018.05.010, PMID: 29803708

[37] Sugita K Kabashima K. Tight junctions in the development of asthma, chronic rhinosinusitis, atopic dermatitis, eosinophilic esophagitis, and inflammatory bowel diseases. J Leukoc Biol. (2020) 107:749–62. doi: 10.1002/JLB.5MR0120-230R, PMID: 32108379

[38] Diesner SC Bergmayr C Pfitzner B Assmann V Krishnamurthy D Starkl P. A distinct microbiota composition is associated with protection from food allergy in an oral mouse immunization model. Clin Immunol. (2016) 173:10–8. doi: 10.1016/j.clim.2016.10.009, PMID: 27789346 PMC5464391

[39] Ma TY Iwamoto GK Hoa NT Akotia V Pedram A Boivin MA. TNF-α-induced increase in intestinal epithelial tight junction permeability requires NF-κB activation. Am J Physiol Gastrointest Liver Physiol. (2004) 286:G367–76. doi: 10.1152/ajpgi.00173.2003, PMID: 14766535

[40] Moon HW. Comparative histopathology of intestinal infections. Adv Exp Med Biol. (1997) 412:1–19. doi: 10.1007/978-1-4899-1828-4_1, PMID: 9191985

[41] Furuya S Furuya K. Chapter three - roles of substance P and ATP in the subepithelial fibroblasts of rat intestinal villi. Int Rev Cell Mol Biol. (2013):133–89. doi: 10.1016/B978-0-12-407696-9.00003-8 23809436

[42] Yang SW Yu M. Role of goblet cells in intestinal barrier and mucosal immunity. J Inflamm Res. (2021) 14:3171–83. doi: 10.2147/JIR.S318327, PMID: 34285541 PMC8286120

[43] McDermaid A Monier B Zhao J Liu BQ Ma Q. Interpretation of differential gene expression results of RNA-seq data: review and integration. Brief Bioinform. (2018) 20:2044–54. doi: 10.1093/bib/bby067, PMID: 30099484 PMC6954399

[44] Lee J-H Yang Y-H Lin Y-T Wang L-C Yu H-H Hu Y-C. Characterizing non-T2 asthma: key pathways and molecular implications indicative of attenuated Th2 response. Inflammation. (2024). doi: 10.1007/s10753-024-02159-3 39466498

[45] Chen MY Su Q Shi YG. Molecular mechanism of IgE-mediated FcεRI activation. Nature. (2025) 637:453–60. doi: 10.1038/s41586-024-08229-8, PMID: 39442557

[46] Ekstedt S Tufvesson E Bjermer L Kumlien Georén S Cardell LO. A new role for “eat me” and “don’t eat me” markers on neutrophils in asthmatic airway inflammation. Allergy. (2020) 75:1510–12. doi: 10.1111/all.14179, PMID: 31919855

[47] Shevchenko M Servuli E Albakova Z Kanevskiy L Sapozhnikov A. The role of heat shock protein 70 kDa in asthma. JAA. (2021) 13:757–72. doi: 10.2147/JAA.S288886, PMID: 33447061 PMC7801907

[48] Zhang HH Wei R Yang XY Xu L Jiang HC Li MK. AMFR drives allergic asthma development by promoting alveolar macrophage–derived GM-CSF production. J Exp Med. (2022) 219:e20211828. doi: 10.1084/jem.20211828, PMID: 35333296 PMC8961293

[49] Wu D Li SQ Liu X Xu JN Jiang A Zhang Y. Alpinetin prevents inflammatory responses in OVA-induced allergic asthma through modulating PI3K/Akt/NF-κB and HO-1 signaling pathways in mice. Int Immunopharmacol. (2020) 89:107073. doi: 10.1016/j.intimp.2020.107073, PMID: 33039967

[50] Rana TS Bansode RR Williams LL. Anti-allergic and anti-inflammatory signaling mechanisms of natural compounds/extracts in in vitro system of RBL-2H3 cell: a systematic review. Cells. (2024) 13:1389. doi: 10.3390/cells13161389, PMID: 39195277 PMC11353167

[51] Falcone FH Wan D Barwary N Sagi-Eisenberg R. RBL cells as models for in vitro studies of mast cells and basophils. Immunol Rev. (2018) 282:47–57. doi: 10.1111/imr.12628, PMID: 29431208

[52] Passante E Ehrhardt C Sheridan H Frankish N. RBL-2H3 cells are an imprecise model for mast cell mediator release. Inflamm Res. (2009) 58:611–8. doi: 10.1007/s00011-009-0028-4 19301096

[53] Opitz C Schade G Kaufmann S Di Berardino M Ottiger M Grzesiek S. Rapid determination of general cell status, cell viability, and optimal harvest time in eukaryotic cell cultures by impedance flow cytometry. Appl Microbiol Biotechnol. (2019) 103:8619–29. doi: 10.1007/s00253-019-10046-3, PMID: 31396681

[54] Harada A Sekido N Akahoshi T Wada T Mukaida N Matsushima K. Essential involvement of interleukin-8 (IL-8) in acute inflammation. J Leukoc Biol. (1994) 56:559–64. doi: 10.20935/AcadMatSci7499 7964163

[55] Yang H Rao JN Wang J-Y. Posttranscriptional regulation of intestinal epithelial tight junction barrier by RNA-binding proteins and microRNAs. Tissue Barriers. (2014) 2:e28320. doi: 10.4161/tisb.28320, PMID: 24843843 PMC4022605

[56] Tsukita S Tanaka H Tamura A. The Claudins: from tight junctions to biological systems. Trends Biochem Sci. (2019) 44:141–52. doi: 10.1016/j.tibs.2018.09.008, PMID: 30665499

[57] Fuentes J de Camargo AC Atala E Gotteland M Olea-Azar C Speisky H. Quercetin oxidation metabolite present in onion Peel protects Caco-2 cells against the oxidative stress, NF-kB activation, and loss of epithelial barrier function induced by NSAIDs. J Agric Food Chem. (2021) 69:2157–67. doi: 10.1021/acs.jafc.0c07085, PMID: 33591188

[58] Roselli M Maruszak A Grimaldi R Harthoorn L Finamore A. Galactooligosaccharide treatment alleviates DSS-induced colonic inflammation in Caco-2 cell model. Front Nutr. (2022) 9:862974. doi: 10.3389/fnut.2022.862974, PMID: 35495925 PMC9047546

[59] Song YL Wang ZG Jiang JZ Piao YH Li L Xu C. DEK-targeting aptamer DTA-64 attenuates bronchial EMT-mediated airway remodelling by suppressing TGF-β1/Smad, MAPK and PI3K signalling pathway in asthma. J Cell Mol Med. (2020) 24:13739–50. doi: 10.1111/jcmm.15942, PMID: 33124760 PMC7754001

[60] Zhao M Zhou A Xu L Zhang X. The role of TLR4-mediated PTEN/PI3K/ Akt /NF-κB signaling pathway in neuroinflammation in hippocampal neurons. Neuroscience. (2014) 269:93–101. doi: 10.1016/j.neuroscience.2014.03.039, PMID: 24680857

